# Cow manure application effectively regulates the soil bacterial community in tea plantation

**DOI:** 10.1186/s12866-020-01871-y

**Published:** 2020-07-01

**Authors:** Shuning Zhang, Litao Sun, Yu Wang, Kai Fan, Qingshan Xu, Yusheng Li, Qingping Ma, Jiguo Wang, Wanming Ren, Zhaotang Ding

**Affiliations:** 1 Tea Research Institute, Qingdao Agricultural University, Qingdao, 266109 Shandong China; 2 College of Horticulture, Northwest A & F University, Yangling, 712100 Shanxi China; 3 Fruit and Tea Technology Extension Station, Jinan, 250000 Shandong China; 4 College of Agriculture, Liaocheng University, Liaocheng, 252000 Shandong China; 5 Rizhao Agricultural Technology Service Center, Rizhao, 276800 Shandong China; 6 Modern Agricultural And Rural Development Research Center Of Shandong Province, Jinan, 250100 Shandong China

**Keywords:** *Camellia sinensis* (L.) O. Kuntze, Cow manure, Bacterial community, Tea plantation soil

## Abstract

**Background:**

Cow manure is not only an agricultural waste, but also an organic fertilizer resource. The application of organic fertilizer is a feasible practice to mitigate the soil degradation caused by overuse of chemical fertilizers, which can affect the bacterial diversity and community composition in soils. However, to our knowledge, the information about the soil bacterial diversity and composition in tea plantation applied with cow manure fertilization was limited. In this study, we performed one field trial to research the response of the soil bacterial community to cow manure fertilization compared with urea fertilization using the high-throughput sequencing technique of 16S rRNA genes, and analyzed the relationship between the soil bacterial community and soil characteristics during different tea-picking seasons using the Spearman’s rank correlation analysis.

**Results:**

The results showed that the soil bacterial communities were dominated by *Proteobacteria, Bacteroidetes, Acidobacteria* and *Actinobacteria* across all tea-picking seasons. Therein, there were significant differences of bacterial communities in soils with cow manure fertilization (CMF) and urea fertilization (UF) in three seasons: the relative abundance of *Bacteroidetes* in CMF was significantly higher than that in UF and CK in spring, and the relative abundance of *Proteobacteria* and *Bacteroidetes* in CMF was significantly higher than that in UF and CK in autumn. So, the distribution of the dominant phyla was mainly affected by cow manure fertilization. The diversity of bacterial communities in soils with cow manure fertilization was higher than that in soils with urea fertilization, and was the highest in summer. Moreover, soil pH, OM and AK were important environmental properties affecting the soil bacterial community structure in tea plantation.

**Conclusions:**

Although different fertilizers and seasons affect the diversity and structure of soil microorganisms, the application of cow manure can not only improve the diversity of soil bacteria, but also effectively regulate the structure of soil bacterial community in tea plantation. So, cow manure fertilization is more suitable for tea plantation.

## Background

Tea (*Camellia sinensis*) is a perennial economic plant in China. Tea cultivation demands more nitrogen for high yield and quality component [1, 2]. However, in order to maximize the tea yields, large amount of chemical fertilizers had been applied in tea plantation. Excessive use of chemical fertilizers could bring passive impacts on the ecological functions and biochemical characteristics in soils, including soil nutrient losses and soil acidification [3, 4]. Therein, soil acidification not only could lead to the deficiencies in phosphorus (P), potassium (K), and magnesium (Mg) nutrients, but also could increase the content of heavy metals in tea leaves [5–7]. Thus, the substitution of chemical fertilizers by organic fertilizers was urgently promoted to mitigate the negative effects of chemical fertilizer overuses. Organic fertilizer played an important role in soil improvement by continuously providing organic matters and available nutrients, which obtained the standard of ecological agriculture [8]. Compared with chemical fertilizer, organic fertilization in tea plantation could improve soil fertility and accomplish carbon accumulation, which was a key factor in determining soil properties and productivity [9, 10].

Soil bacteria occupied an irreplaceable position in the function and sustainability of agro-ecosystems on account of their contributions to soil fertility and nutrient cycling [11–13]. They responded more quickly to changes in the environment than to chemical or physical properties, resulting in dynamic changes in microbial biomass, activity, diversity, and composition [14]. Significant differences in microbial biomass and microbial diversity have been observed in tea plantation soils with chemical and organic fertilization. Long-term tea cultivation with chemical fertilizers altered the bacterial composition of soil and decreased microbial metabolic activity, resulting in a reduction of beneficial bacteria [15, 16]. In contrary, organic fertilizer increased the soil microbial diversity, altered the network structure and improved potential ecosystem function [17, 18].

As a kind of agricultural waste, cow manure could be used in tea plantation as organic fertilizer. However, to our knowledge, the study information about soil bacterial communities of tea plantation with cow manure fertilization was limited. In the present study, to research the effects of cow manure on soil bacterial diversity and composition in tea plantation, we performed one field trial (unfertilized, urea and cow manure fertilization) during different tea-picking seasons. The diversity and composition of soil bacterial community were analyzed using a high-throughput sequencing technique of 16S rRNA genes, and the relationships between soil bacterial communities and soil parameters were also analyzed in spring, summer and autumn. The objectives of the study were: 1) to reveal the variations of diversity and composition in soil bacteria with cow manure and 2) to compare the changes of soil bacterial communities in spring, summer and autumn. This study not only proved that the application of cow manure can effectively control the soil bacterial community, but also provided an important theoretical basis for the rational application of organic fertilizer in tea plantation.

## Results

### The diversity of soil bacterial community

To annotate and evaluate the bacterial communities in soils, we conducted the optimized sequences using the categorical operation. Rarefaction curve analysis showed that each curve was close to flat finally, indicating that the sample size of this sequencing was sufficient and the data of sequencing was reasonable (Fig. S1). The numbers of OTUs in CMF were invariably higher than that in UF, manifesting that the application of cow manure increased the numbers of OTUs (Fig. S2). With a 3% dissimilarity threshold, there were 1130 OTUs, 1430 OTUs and 722 OTUs in spring, summer and autumn, respectively.

To quantify the diversity and richness of bacterial community of soils among three treatments, we analyzed α-diversity index using random sampling method (Table 1). The results showed that the different fertilizer treatments and different seasons affected the diversity of bacterial communities. The richness of CMF was significantly lower than that of CK and UF in the spring, but in the autumn, the richness of CMF was significantly higher than that of CK and UF. In addition, the richness and evenness were also affected by different seasons. The Chao1 and the Shannon indices both increased from spring to summer, but decreased from summer to autumn. Thus, soil bacterial communities had high diversity in summer, and cow manure fertilization greatly affected the diversity of bacterial communities in soils.

**Table 1:** The diversity indices of soil bacterial communities

Season	Sample	ACE	Chao1	Shannon	Simpson
spring	CK	962.27 ± 56.61a	977.33 ± 58.23a	8.07 ± 0.42a	0.99 ± 0.01a
	UF	891.56 ± 59.70ab	912.37 ± 54.88ab	7.88 ± 0.42a	0.98 ± 0.01a
	CMF	736.35 ± 256.95b	743.02 ± 241.83b	6.90 ± 0.83b	0.97 ± 0.01a
summer	CK	1061.41 ± 81.53a	1091.28 ± 75.89a	8.26 ± 0.32a	0.99 ± 0.01a
	UF	1033.05 ± 37.49a	1048.23 ± 45.50a	7.98 ± 0.27a	0.98 ± 0.01a
	CMF	1042.33 ± 91.99a	1041.37 ± 113.25a	7.81 ± 0.72a	0.98 ± 0.01a
autumn	CK	539.53 ± 8.16c	550.50 ± 10.63c	7.46 ± 0.28a	0.98 ± 0.01a
	UF	571.62 ± 14.20b	582.82 ± 14.55b	7.46 ± 0.24a	0.98 ± 0.01a
	CMF	610.96 ± 26.77a	617.55 ± 27.17a	7.57 ± 0.28a	0.99 ± 0.00a

The mean value ± standard deviation (*n* = 6). Values with the same letter are not significantly different (*p* < 0.05)

*CK* control experiment, *UF* urea fertilization, *CMF* cow manure fertilization

**Fig. 1:**
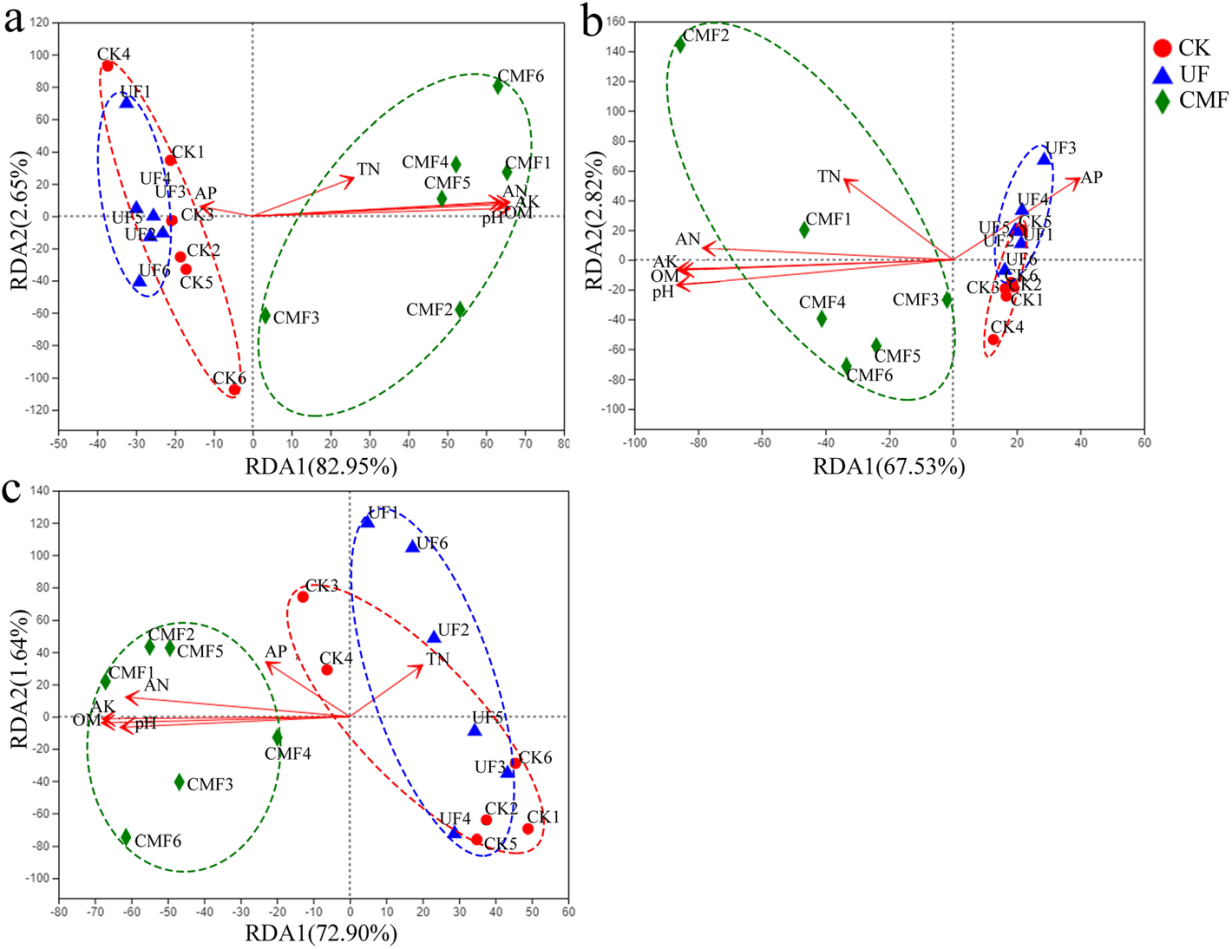
The Redundancy analysis (RDA) of bacterial communities in soils with different fertilizations. The number of soil bacterial OTUs in spring (**a**), summer (**b**) and autumn (**c**). CK: unfertilized; UF: urea fertilization; CMF: cow manure fertilization

To observe the relevant factors of the distribution of bacterial communities, we performed the redundancy analysis (RDA) using direct gradient analysis technique (Fig. 1). Three treatments clustered closely in spring, summer and autumn, except several uncontrolled one. The first principal coordination axis accounted for 82.95, 67.53 and 72.90% in three seasons, respectively. There were significant differences between treatments with or without cow manure. The second principal coordination axis explained 2.65, 2.82 and 1.64% in three seasons, respectively. Obviously, the second major factor had little effect on CMF. The fertilizations with or without cow manure were in two branches separately. The distribution of bacterial communities was mainly affected by cow manure. In addition, redundancy analysis showed that AN, AK, OM and pH were important environmental factors affecting the distribution of soil samples.

### The composition of soil bacterial community

**Fig. 2:**
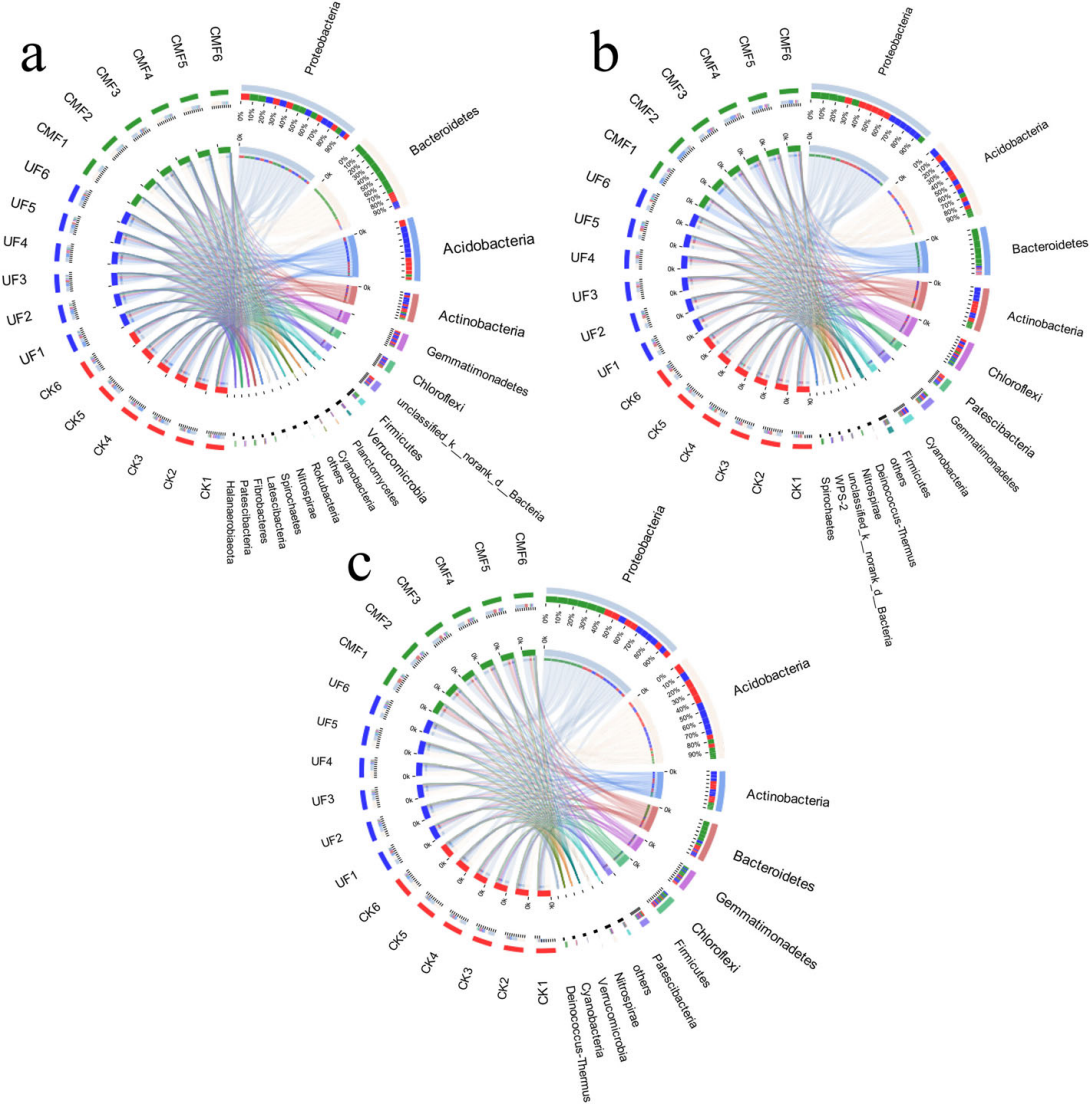
The composition of bacterial community in soils under different fertilizations. The distribution of core bacterial phyla in spring (**a**), summer (**b**) and autumn (**c**). CK: unfertilized; UF: urea fertilization; CMF: cow manure fertilization

**Fig. 3:**
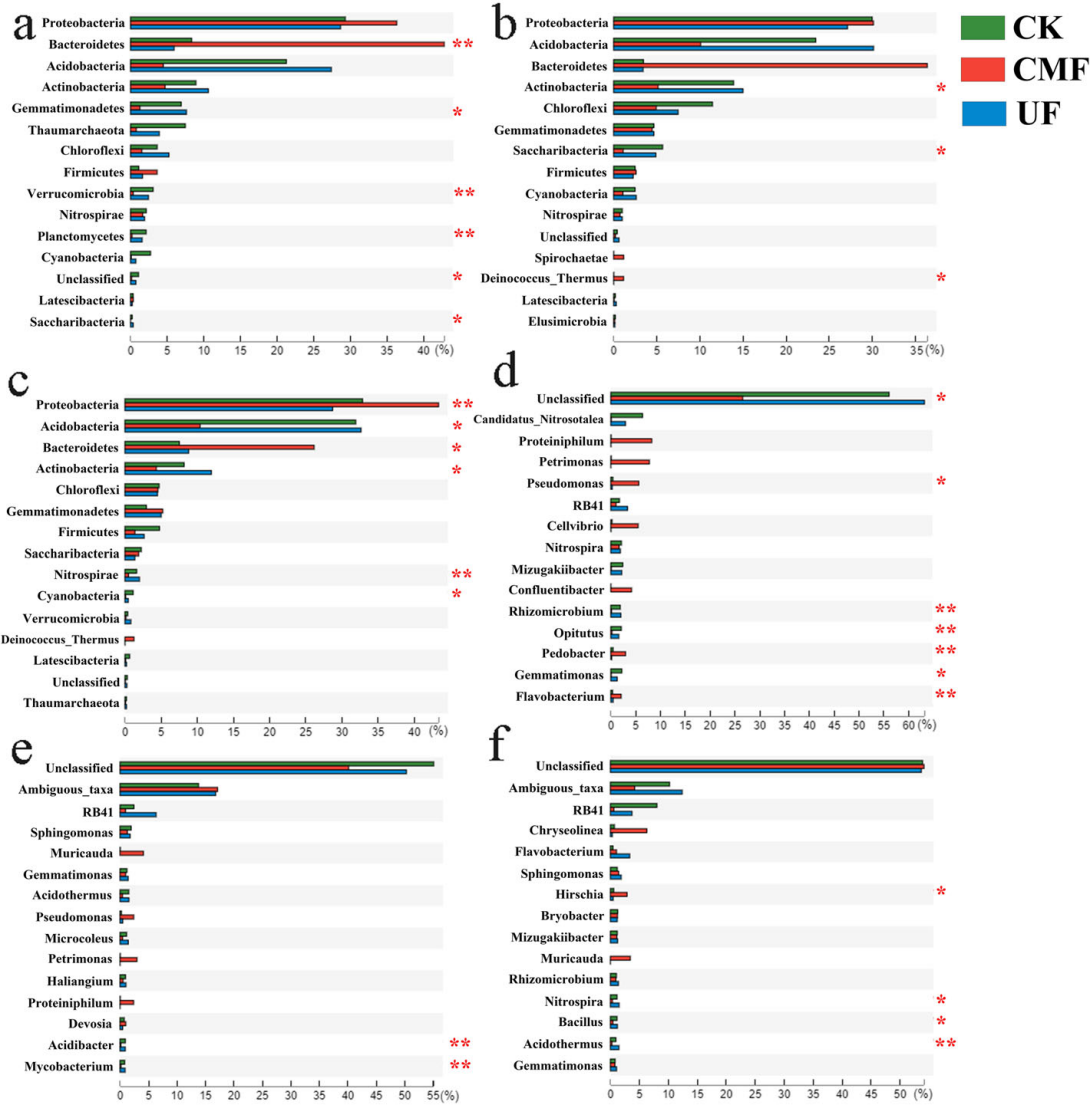
The relative abundance of core bacterial community in soils with different fertilizations. The relative abundance of soil bacterial community with significant different at phylum level in spring (**a**), summer (**b**) and autumn (**c**). The relative abundance of soil bacterial community with significant different at genus level in spring (**d**), summer (**e**) and autumn (**f**). CK: unfertilized; UF: urea fertilization; CMF: cow manure fertilization. Asterisks indicate significantly different values: *p < 0.05, **p < 0.01

To observe the composition of the soil bacterial community, we aligned the top30 OTUs with the SILVA 119 database and dissected the composition of community at the phylum level (Fig. 2). Species analysis showed that the dominant bacterial communities in soils mainly derived from four phyla, including *Proteobacteria, Bacteroidetes, Acidobacteria* and *Actinobacteria,* the other phyla only occupied a small portion of all phyla. *Proteobacteria* dominated the entire bacterial communities regardless of seasons and treatments, accounting for 30.3, 30.69 and 33.15%. The abundance of *Acidobacteria* was increased in CMF from spring to autumn, accounting for 3.09, 9.87 and 12.13%. The ratios of *Proteobacteria* to *Acidobacteria* (P/A) which could reflect the soil nutrition status were 17.85, 4.83 and 4.08 in CMF from spring to autumn, and the P/A ratios were 1.16, 1.05, 0.92 in UF (Table S1). *Proteiniphilum*, *Fermentimonas, Chujaibacter* and *Pseudomonas* were the dominant bacterial genus in spring (Fig. S3a), *RB41*, *Sphingomonas, MND1* and *Muricauda* were the dominant bacterial genus in summer (Fig. S3b), and *RB41, Chujaibacter, MND1* and *Chryseolinea* were the dominant bacterial genus in autumn (Fig. S3c). In addition, there were significant differences in soils with cow manure fertilization and urea fertilization (Fig. 3). At the phylum level, *Bacteroidetes*, *Verrucomicrobia* and *Planctomycetes* presented significant differences in spring, and *Actinobacteria*, *Saccharibacteria* and *Deinococcus_Thermus* presented significant differences in summer, and *Proteobacteria*, *Acidobacteria*, *Bacteroidetes*, *Actinobacteria* and *Nitrospirae* presented significant differences in autumn. Therein, the relative abundance of *Bacteroidetes* in CMF was significantly higher than that in UF and CK in spring, and the relative abundance of *Proteobacteria* and *Bacteroidetes* in CMF was significantly higher than that in UF and CK in autumn. At the genus level, *Rhizomicrobium*, *Opitutus*, *Pedobacter* and *Flavobacterium* presented significant differences in spring, and *Acidibacter* and *Mycobacterium* presented significant differences in summer, and *Hirschia*, *Nitrospira*, *Bacillus* and *Acidothermus* presented significant differences in autumn. Therein, the relative abundance of *Pedobacter, Pesudomonas* and *Flavobacterium* in CMF was significantly higher than that in UF and CK in spring, and the relative abundance of *Hirschia* presented significant differences in autumn.

### Relationships between the preponderant phyla of soil bacteria and the soil physicochemical characteristics

**Fig. 4:**
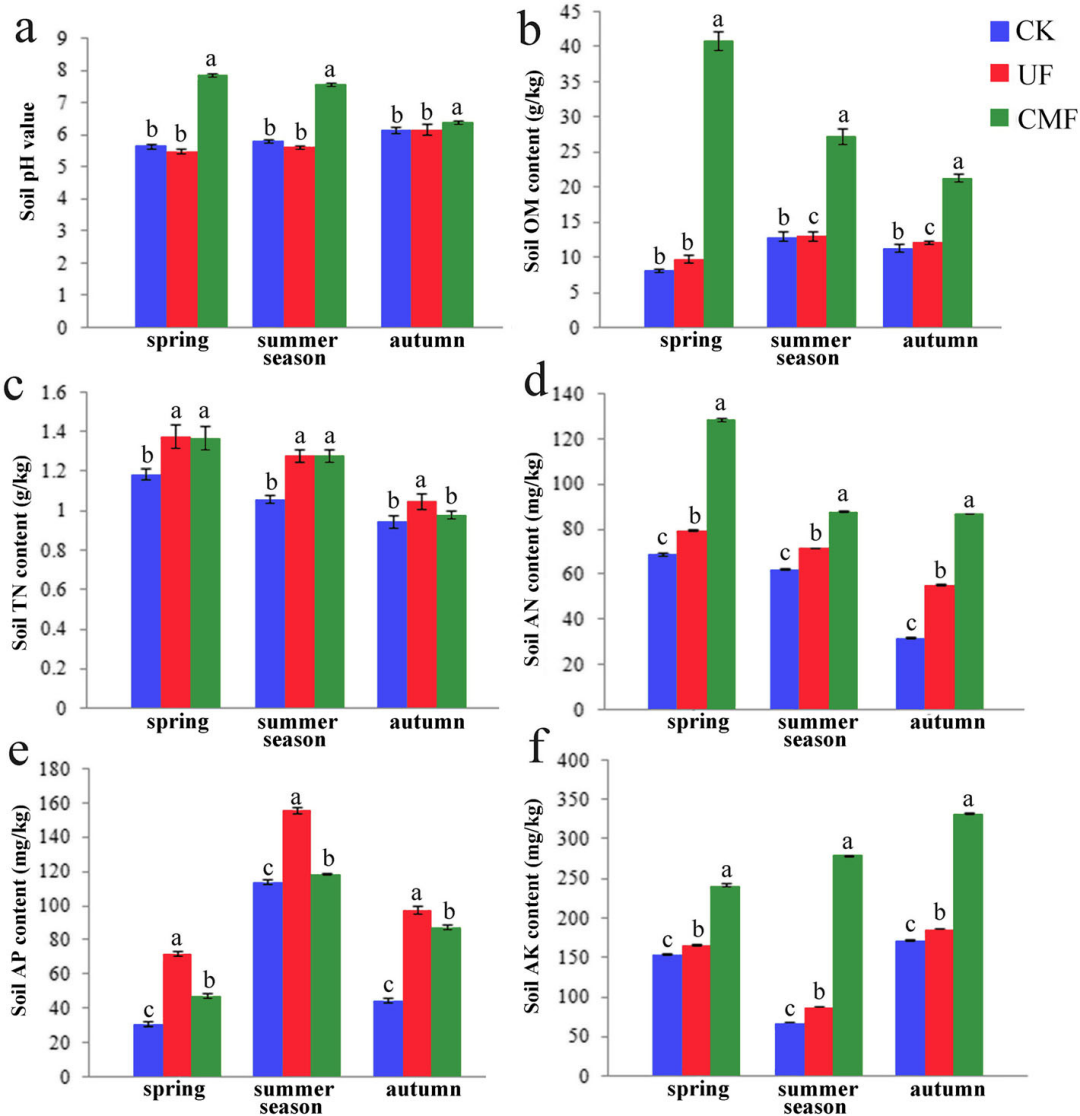
The physicochemical characteristics of soils with different fertilizations. **a** The pH value of soils in spring, summer and autumn. The OM (**b**), TN (**c**), AN (**d**), AP (**e**) and AK (**f**) contents of soils in spring, summer and autumn. CK: unfertilized; UF: urea fertilization; CMF: cow manure fertilization

To evaluate the physicochemical characteristics of soils with different fertilizations, we analyzed the contents of soil nutrients in three seasons (Fig. 4). The data revealed significant difference between the soils with cow manure fertilization and the soils with urea fertilization. The results showed that the pH in soil with cow manure fertilization was significantly higher than that in soil with unfertilized and urea fertilization. Soil TN contents did not fluctuate significantly from spring to autumn, but among three treatments, the TN contents in CMF and UF were significantly higher than those in CK. The AP contents in CMF were lower than that in UF. Compared with UF, the application of cow manure significantly increased the contents of AN, AK and OM in three seasons.

**Fig. 5:**
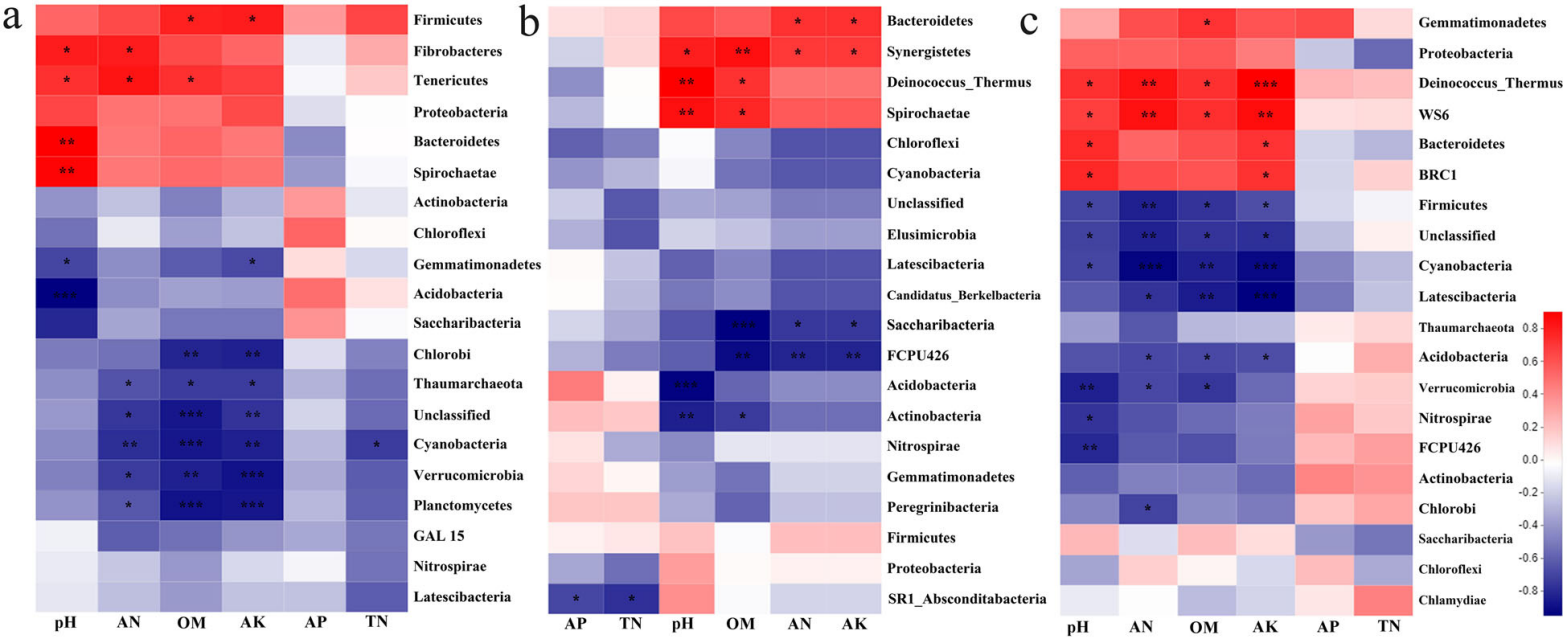
The spearman correlation heatmap between soil physicochemical characteristics and bacterial communities in soils with different fertilizations. The relationships between soil physicochemical characteristics and bacterial communities in spring (**a**), summer (**b**) and autumn (**c**)

To study the impacts of soil environmental properties on the abundances of soil bacterial communities, we analyzed the relationships between soil bacterial phyla and soil physicochemical properties in three seasons using spearman correlation heat map (Fig. 5a-c). In spring, the soil pH content was positively correlated with the abundances of *Bacteroidetes* and *Spirochaetae*, and was negatively correlated with the abundance of *Acidobacteria*. The soil OM and AK contents were positively correlated with the abundances of *Firmicutes*, and were negatively correlated with the abundances of *Chlorobi*, *Cyanobacteria* and *Planctomycetes*. In summer, the soil pH content was positively correlated with the abundance of *Spirochaetae*, and was negatively correlated with the abundances of *Acidobacteria* and *Actinobacteria*. The soil OM content was positively correlated with the abundances of *Synergistetes*, and was negatively correlated with the abundances of *Actinobacteria* and *Saccharibacyeria*. The soil AN and AK contents were positively correlated with the abundance of *Bacteroidete*. In autumn, soil pH value had significant correlation with high abundance phyla. The soil OM, AN and AK contents were positively related to the abundances of *Acidobacteria*, *Firmicutes*, *Cyanobacteria and Latescibacteria*. So, soil pH, OM and AK were important environmental properties affecting the soil bacterial community structure in tea plantation.

## Discussion

The diversity of soil bacterial community was critical to the integrity, stability and sustainability of soil ecosystems [19]. Different fertilization treatments could influence soil microenvironment in agro-ecosystems. The negative effect of chemical fertilization on soil bacterial diversity across different agricultural ecosystems has been reported [20, 21]. A significant decrease in bacterial diversity was at high nitrogen rate, which could create stressful conditions and inhibit bacterial growth [22]. On the contrary, bacterial diversity in soils with organic fertilization was significantly higher than that in soils with chemical fertilization of legume systems and wheat systems [23, 24]. Similarly, a field experiment in tea plantation was conducted to study the effects of different fertilization on soil microbial communities, showing that organic manure treatment had the higher diversity of soil bacterial community [9]. However, previous studies only focused on the impact of different organic matters on bacterial diversity, but did not consider the impact of soil bacterial diversity in tea-picking seasons. In the present study, we examined the dynamic changes of soil bacterial diversities in tea plantation applied with cow manure fertilization and urea fertilization in three different seasons. The results showed that the diversity of soil bacteria under both fertilization treatments changed in similar trends: a significant increase from spring to summer and a significant decrease from summer to autumn. Wang et al. (2018) demonstrated that temperature was the major environmental factor impacting the bacterial community in cow manure composting progress [25]. So, we concluded that it is possible that the activities of bacteria gradually increased with the increase of temperature from spring to summer, resulting in the higher diversity of soil bacteria. While with the decrease of temperature from summer to autumn, the activities of bacteria gradually decreased, resulting in the lower diversity of soil bacteria. Meanwhile, the soil with cow manure had a higher pH in spring, but in autumn, the pH decreased. We considered that pH and temperature were important factors determined the diversity of soil bacterial communities. Moreover, in the present study, the bacterial diversity of soil under cow manure fertilization with Chao1 index of 617.55 was significantly higher than that in soil under urea fertilization with Chao1 index of 582.82 in autumn. The full utilization of nutrients in cow manure by soil bacteria might be an important reason for high diversity in autumn. Therefore, we think that the seasonal change, like fertilization, will cause the change of soil bacterial diversity in tea plantation. Season and fertilization might work together on the diversity of soil microorganism.

The structure of soil microbial communities was sensitive to different fertilization treatments in agro-ecosystems. The most frequent phyla were *Proteobacteria*, *Chloroflexi*, *Acidobacteria*, *Actinobacteria* and *Bacteroidetes* in agricultural soils [13, 26]. In tea plantation, *Acidobacteria, Proteobacteria, Actinobacteria*, and *Chloroflexi* were the most abundant phyla in soils with different fertilization [17]. In the present study, the composition of soil bacterial community were dissected at the phylum level, and the results showed that the preponderant phyla of soil bacteria were *Proteobacteria*, *Acidobacteria, Bacteroidetes* and *Actinobacteria* in tea plantation. Identifying difference of soil bacteria in different fertilized and unfertilized soil could provide more insights into the evaluation of soil fertility and the effect of organic fertilizer.

*Proteobacteria* and *Acidobacteria* are the two important phyla in the microbial community. High abundances of *Proteobacteria* and *Acidobacteria* could enhance cycling of essential nutrients, which could improve soil fertility and sustainable utilization. *Proteobacteria* were adapted to the environment with abundant resources [27]. *Acidobacteria* were generally considered to like oligotrophic environments which resource limited [28]. Smit et al. (2001) and Torsvik & Øvreås (2002) reported that the ratio of *Proteobacteria* to *Acidobacteria* could reflect the soil nutrition status: A higher ratio means that more organic matter input [29, 30]. Ji et al. (2018) reported that higher relative abundance of *Proteobacteria* correlated positively with the organic substitution ratio in tea plantation, but the abundance of *Acidobacteria* was inversely correlated with this ratio [17]. However, there is no information about the feature of P/A in tea plantation with cow manure. In the present study, the abundances of *Proteobacteria* significantly increased in soils with cow manure fertilization, but with urea fertilization, the abundances of *Proteobacteria* decreased. The P/A in soils with cow manure was higher than that in soils with urea, indicating that the soil with cow manure could offer a preferred habitat with an intermediate level between the copiotrophic and oligotrophic conditions for soil bacteria. *Actinobacteria* and *Bacteroidetes* are important phyla of bacteria in soil and manure, respectively*.* It was known that *Actinobacteria* were functionally diverse and contributed to the decomposition of organic matter [31]. They were relatively ineffective competitors under high nutrient conditions versus other bacteria, which they occupied an inferior position compared with *Proteobacteria* or *Acidobacteria*. But their slow growth and ability to break down complex substrates gave them a competitive advantage over other bacteria [1]. In the study, the relative abundance of *Actinobacteria* in soils with urea (12.64%) was significantly higher than that of in soils with cow manure (4.4%), indicating that the application of urea created a relatively nutrient-poor environment over cow manure that gave the *Actinobacteria* a lot of advantages. The results were consistent with the former studies [23, 31, 32]. *Bacteroidetes* are the main microbial species in the feces*. Bacteroidetes* were found to be predominant in the cow manure composting [33]. Pervious study showed that *Bacteroidetes* were observed to be predominant in the cow manure composting system, and they had high degradation capacity oncellulose in the thermophilic phase of composting [25]. However, the *Bacteroidetes* were not found to be the dominant bacteria in soils applied organic fertilizer. In the present study, the relative abundance of *Bacteroidetes* (33.69%) in soils with cow manure was significantly higher than that of in soils with urea (5.35%), indicating that the soil in tea plantation with cow manure was suitable for the growth of *Bacteroidetes*. They might actively participate in a series of activities in soils and make an important contribution to soil nutrient conversion and material cycling. *Flavobacterium* belonged to *Bacteroidetes,* and the relative abundance of *Flavobacterium* was significantly enriched in soils with cow manure fertilization (Fig. 3). It had been found that *Flavobacterium* could degrade macromolecular organic matter such as protein and lipid, and had certain nitrification and potential denitrification ability [34]. The bacteria in the genus *Pesudomonas* and *Flavobacterium* were aerobic denitrifiers, and could participate in the action of aerobic denitrification, which was the key of natural nitrogen cycling [35]. High relative abundance of *Pesudomonas* and *Flavobacterium* in soils with cow manure fertilization indicated that the organic fertilization could enrich bacteria to involve in the nitrogen cycle and promote the biochemical cycle in tea plantation.

Exogenous bacterial input through the application of manures could increase soil bacterial diversity, causing the alteration in bacterial composition [36]. Soil environmental factors played important roles in changing the structure of soil bacterial communities [37]. Soil pH was a critical factor for bacterial diversification and exerted a strong influence on the structure of soil microbial communities [38, 39]. Previous study showed that the direct influence of pH on bacterial community composition was probably due to the narrow pH ranges for optimal growth of bacteria, showing that a negative correlation was the relative abundance of *Acidobacteria* increased with lower pH [40]. This effect was also found in tea plantation: soil pH played the most important role in shaping the bacterial community structure, showing that *Proteobacteria* and *Actinobacteria* both significantly correlated with pH [17]. In the present study, urea fertilization decreased the soil pH, while cow manure fertilization significantly increased the soil pH, indicating that adding organic matter could mitigate the soil acidification caused by chemical fertilization. The alleviation of soil acidification by organic matter application could be attributed to the alkaline matter in the cow manure, which could neutralize the soil acidity. In addition, the relative abundance of *Acidobacteria* was negatively correlated with pH, indicating that *Acidobacteria* was most sensitive to the change of soil pH. The carbon source from soil organic matter was considered as an important factor influencing the bacterial community composition in previous studies [41]. In the present study, soil OM content had significant correlation with soil bacterial phyla, indicating that cow manure provides rich carbon source to promote the activity of soil bacteria. Additionally, soil AP had a strong effect on the bacterial community composition [42]. However, in the present study, no significant effect of AP on the bacterial community was observed. Instead, soil AK had significant effect on soil bacterial community structure. The results indicated that tea plant preferred potassium to phosphorus. Therefore, soil pH, OM and AK could provide comprehensive indexes of soil conditions that directly changed the structure of bacterial communities in different seasons and played important roles in altering the composition of soil bacterial communities. We concluded that soil bacteria could select suitable soil characteristics according to its own characteristics, and the change of soil environment could also affect soil bacteria in tea plantation.

## Conclusions

Our results highlighted the influence of the application of cow manure on soil bacterial diversity and community structure in tea-picking seasons. Compared with urea fertilization, cow manure fertilization significantly increased the diversity of soil bacteria and effectively regulates the structure of soil bacterial communities. Moreover, soil pH, OM and AK were important environmental properties affecting the soil bacterial communities. It provides theoretical support for the rational use of cow manure for tea cultivation, and is of great significance to reduce the amount of chemical fertilizer and protects the soil ecological environment of tea plantation.

## Methods

### Field experiment

The field experiment was conducted at Qingdao Agricultural University in Shandong, China (36° 19′ N, 120° 23′ E, elevation 54.88 m). The average temperature was 25.3 °C and the average annual precipitation was 662.1 mm. The soil of tea field was classified as brown loamy soil. The major soil parameters were as follows: pH 5.6, organic matter (OM, 8.17 g/kg), total nitrogen (TN, 1.18 g/kg), available nitrogen (AN, 68.49 mg/kg), available phosphorus (AP, 26.84 mg/kg) and available potassium (AK, 225.27 mg/kg).

The experiment design included three treatments: CK (control experiment, unfertilized), UF (urea fertilization, N: 46.7%) and CMF (cow manure fertilization, N: 1.5%). All treatments were replicated six times. Each plot was 90 m^2^ and arranged randomly. The tea trees were planted in 2008 with a row spacing of 1.5 m, and plant spacing of 0.33 m, using the tea tree variety ‘Huangshanzhong’. The exact location of fertilization was perpendicular to the bottom of the tree canopy, digging a fertilizer ditch with depth of 20 cm and width of 20 cm, and covered the soil with 5 cm after fertilization. The inputs of cow manures were 20 t/ha. The major cow manure characteristics were as follows: pH 8.4, OM: 71.2%, TN: 1.5%, TP: 0.81%, TK: 0.98%. The total nitrogen contents of UF and CMF stayed the same (300 kg/ha) when the fertilizers were applied to the field by a one-time application in February 25, 2017.

### Soil sampling

We collected soil samples of CK, UF and CMF in tea-peaking seasons: spring (March 27, 2017), summer (June 2, 2017) and autumn (August 4, 2017). The exact location of sampling was in the middle of the fertilizer ditch. Ten soil cores (2 cm in diameter) were collected in each plot to a depth of 20 cm. The soil sampling was homogenized by mixing together and was passed through a 2-mm sieve for discarding mixed above-ground materials (plant residue and stones). Each soil sample was further divided into two parts: one part was frozen quickly in liquid nitrogen, and stored at − 80°Cuntil the microbial communities were analyzed; the other part was dried at room temperature for 7 days and then analyzed for pH, available nitrogen (AN), available phosphorus (AP), available potassium (AK), organic matter (OM), total nitrogen (TN) at the Soil Testing Laboratory.

### DNA extraction and 16S rDNA sequencing

All reactions were carried out in 25 μL (total volume) mixtures containing approximately 250 mg of genomic DNA extract, 12.5 μL PCR Premix, 2.5 μL of each primer, and PCR-grade water to adjust the volume. DNA samples were quantified using a Qubit 2.0Fluorometer (Invitrogen, Carlsbad, CA, USA). 30–50 ng DNA was used to generate ampliconsusing a MetaVx™ Library Preparation kit. V3, V4, and V5 hypervariable regions of prokaryotic 16S rDNA were selected for generating amplicons and following taxonomy analysis. The experimental procedure was conducted as described in a previous study [43]. All primer tests were performed as described in a previous study using standard settings [44]. The V3 and V4 regions were amplified using forward primers containing the sequence “CCTACGGRRBGCASCAGKVRVGAAT” and reverse primers containing the sequence “GGACTACNVGGGTWTCTAATCC”. The V4 and V5 regions were amplified using forward primers containing the sequence “GTGYCAGCMGCCGCGGTAA” and reverse primers containing the sequence “CTTGTGCGGKCCCCCGYCAATTC”. 1st round PCR products were used as templates for 2nd round amplicon enrichment PCR. At the same time, indexed adapters were added to the ends of the 16S rDNA amplicons to generate indexed libraries ready for downstream NGS sequencing on Illumina Miseq. DNA libraries were validated by Agilent 2100 Bioanalyzer (Agilent Technologies, Palo Alto, CA, USA) and quantified by Qubit 2.0 Fluorometer. DNA libraries were multiplexed and loaded on an IlluminaMiSeq instrument according to manufacturer’s instructions (Illumina, San Diego, CA, USA). The QIIME data analysis package was used for 16S rDNA data analysis [45]. The forward and reverse reads were joined and assigned to samples based on barcode and truncated by cutting off the barcode and primer sequence. Quality filtering on joined sequences was performed and sequence which did not fulfill the following criteria were discarded: sequence length < 200 bp, no ambiguous bases, mean quality score > = 20. Then the sequences were compared with the reference database (RDP Gold database) using UCHIME algorithm to detect chimeric sequence, and then the chimeric sequences were removed. The effective sequences were used in the final analysis. Sequences were grouped into operational taxonomic units (OTUs) using the clustering program VSEARCH(1.9.6) against the Silva119 database pre-clustered at 97% sequence identity. The raw sequencing data were deposited in NCBI Sequence Read Archive (SRA) under accession number PRJNA593402 for bacteria.

### Statistical analysis

The means and standard deviations of the data were calculated and statistically examined by ANOVA and Duncan’s multiple range tests using SPSS (SPSS, Inc., Chicago, IL, USA). The significance level was set at *p* < 0.05 unless otherwise stated. The taxonomic alpha diversity indices, such as the Chao1 index and the Shannon index, were calculated using Mothur software (version 1.31.2, *http://www.mothur.org/*). The Circos graph was built using Circos-0.67-7 software (http://circos.ca/) [45]. The Venn diagram was obtained by the tool of Venny (*http://bioinfogp.cnb.csic.es/tools/venny/*). The redundancy analysis (RDA) was obtained using “vegan” in R software. The possible correlation between the relative abundance of bacterial phyla and soil properties were analyzed by performing Spearman’s rank correlation analysis (a non-parametric measure of correlation coefficient) using “ pheatmap package” in R software.

## Supplementary information

**Additional file 1: Figure S1.** The rarefaction curve of soil bacterial communities in spring (a), summer (b) and autumn (c).

**Additional file 2: Figure S2.** The Venn diagram of soil bacterial communities in spring (a), summer (b) and autumn (c).

**Additional file 3: Figure S3.** The relative abundance of soil bacterial community at genus level in spring (a), summer (b) and autumn (c).

**Additional file 4: Table S1.** The relative abundance of the dominant phyla and P/A ratio in soil bacterial communities.

## Data Availability

The raw sequencing data were deposited in NCBI Sequence Read Archive (SRA) under accession number PRJNA593402 for bacteria.

## Abbreviations

AN: Available nitrogen
AP: Available phosphorus
AK: Available potassium
OM: Organic matter
OTU: Operational Taxonomic Units
RDA: Redundancy analysis
RDP: Ribosomal Database Project
TN: Total nitrogen
