# Supplementary figures and images for: Cow manure application effectively regulates the soil bacterial community in tea plantation
Source: BMC Microbiol. 2020 Jul 1;20:190. doi: 10.1186/s12866-020-01871-y (PMC7329415; doi:10.1186/s12866-020-01871-y)

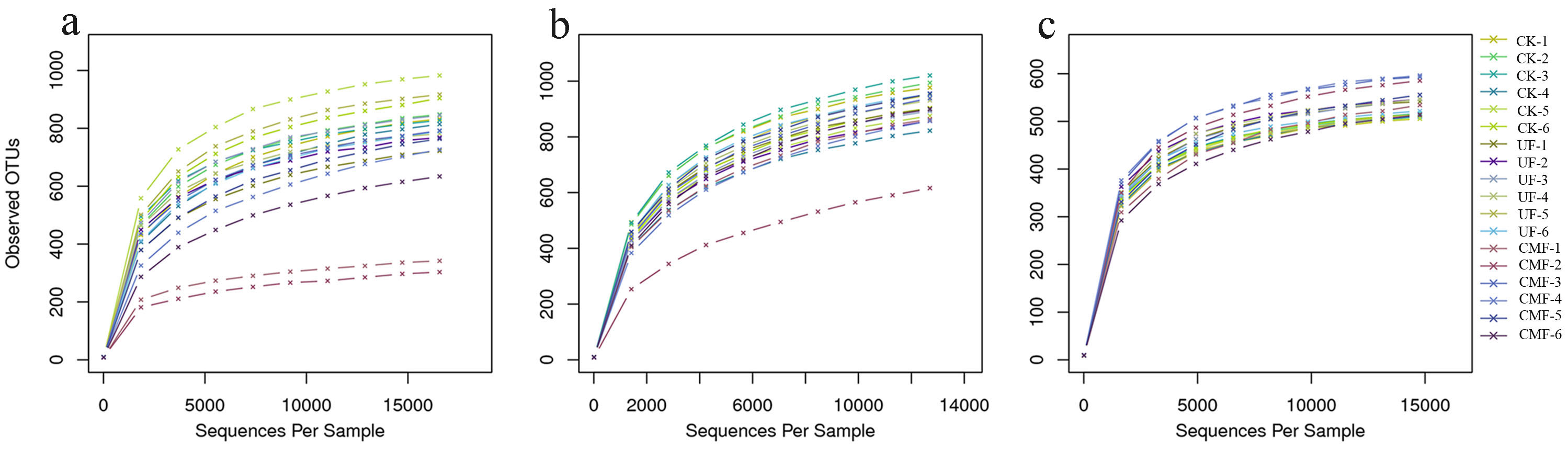

Supplement: Supplementary file 1 — Additional file 1: Figure S1. The rarefaction curve of soil bacterial communities in spring (a), summer (b) and autumn (c). [file 12866_2020_1871_MOESM1_ESM.jpg]

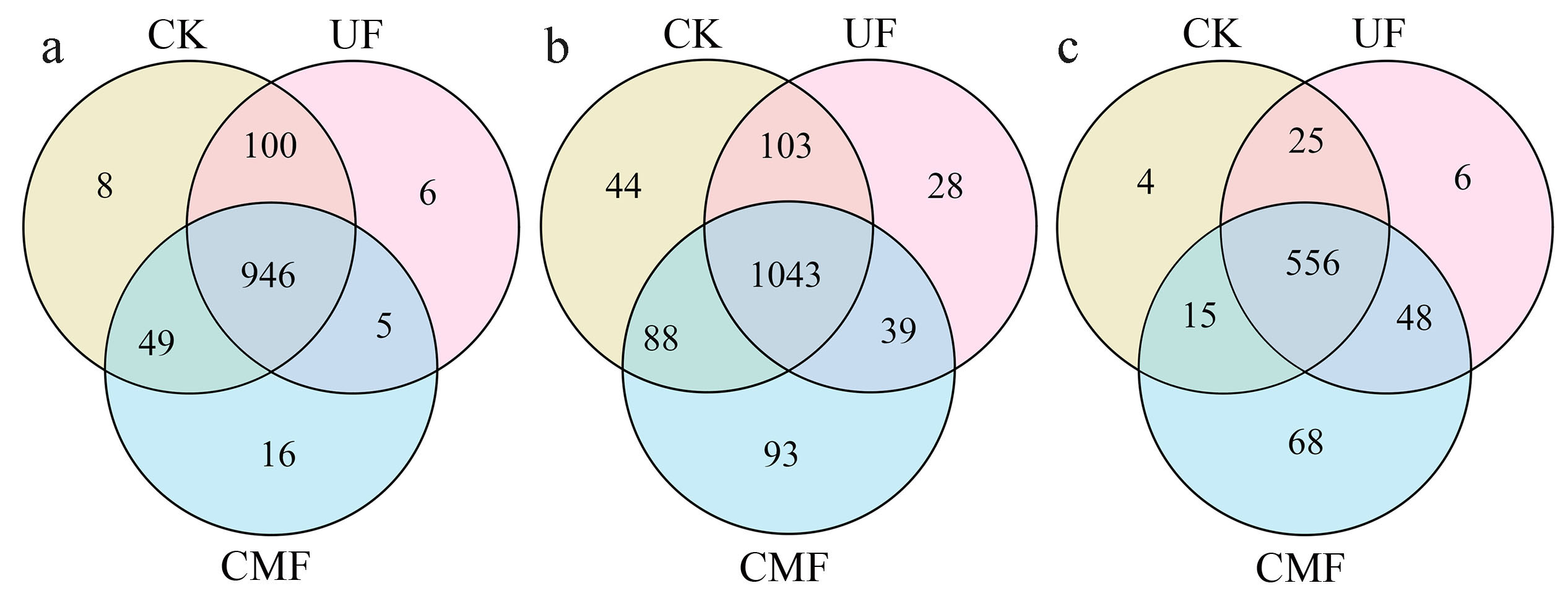

Supplement: Supplementary file 2 — Additional file 2: Figure S2. The Venn diagram of soil bacterial communities in spring (a), summer (b) and autumn (c). [file 12866_2020_1871_MOESM2_ESM.jpg]

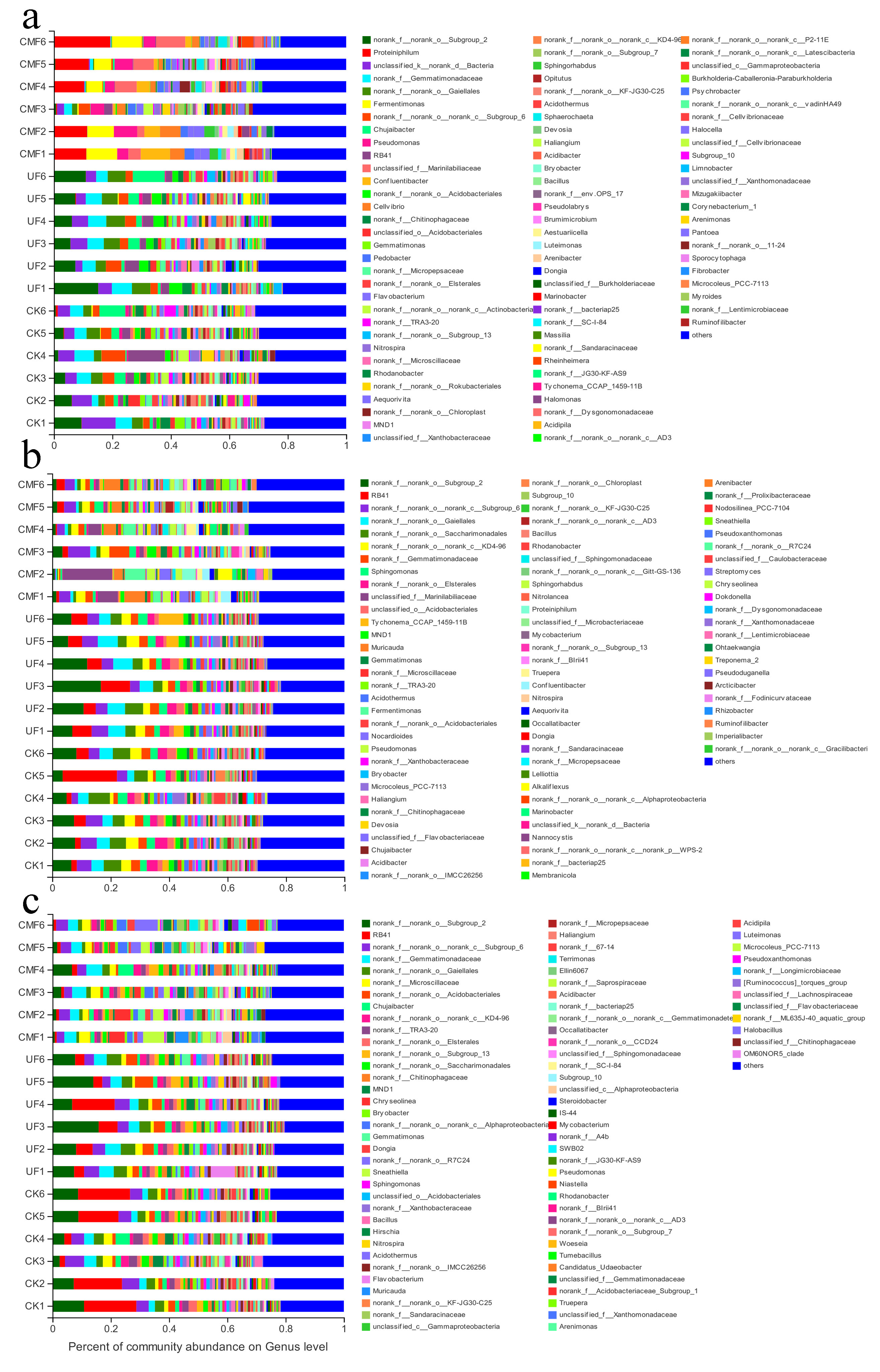

Supplement: Supplementary file 3 — Additional file 3: Figure S3. The relative abundance of soil bacterial community at genus level in spring (a), summer (b) and autumn (c). [file 12866_2020_1871_MOESM3_ESM.jpg]
